# Development of putative probiotics as feed additives: validation in a porcine-specific gastrointestinal tract model

**DOI:** 10.1007/s00253-016-7812-1

**Published:** 2016-09-15

**Authors:** Soyoung Yeo, Suro Lee, Hyunjoon Park, Heuynkil Shin, Wilhelm Holzapfel, Chul Sung Huh

**Affiliations:** 1 Research Institute of Eco-friendly Livestock Science, Institute of Green-Bio Science and Technology, Seoul National University, Pyeongchang, Gangwon-do 232-916 South Korea; 2 Advanced Green Energy and Environment Institute (AGEE), Handong Global University, Pohang, Gyeongbuk 791-708 South Korea; 3 School of Life Sciences, Handong Global University, Pohang, Gyeongbuk 791-708 South Korea; 4 Graduate School of International Agricultural Technology, Seoul National University, Pyeongchang, Gangwon-do 232-916 South Korea

**Keywords:** Probiotics, *Lactobacillus salivarius*, Feed additives, Porcine, Gastrointestinal tract, Adhesion ability

## Abstract

Enforced restrictions on the use of antibiotics as growth promoters (AGPs) in animal production have prompted investigations into alternative feed additives in recent decades. Probiotics are currently the main feed additive used in livestock. However, the selection of probiotic candidates relies on human-based methods and little is known about the verification criteria for host-specific selection. We investigated the probiotic potential of *Lactobacillus salivarius* strains isolated from fed pig feces for their use as porcine feed additives. Two methods were developed that simulated the pig gastrointestinal (GI) tract and the intestinal epithelium, and these were compared with human-based in vitro methods and used for selecting porcine probiotics. *Lactobacillus salivarius* strain LS6 was identified as a promising probiotic strain for potential use as a porcine feed additive. This strain prevented disruption of the epithelial integrity of pig small intestine (PSI) cells by inhibiting the adherence of enterotoxigenic *Escherichia coli* K88. It also showed high survival rates in the in vitro pig GI tract model and good adhesion to PSI cells. We propose that host target-specific screening and validation methods are important tools in the development of effective probiotic feed additives, and this approach may support future-oriented agriculture.

**Electronic supplementary material:**

The online version of this article (doi:10.1007/s00253-016-7812-1) contains supplementary material, which is available to authorized users.

## Introduction

Farm animals are affected by numerous environmental stress factors. In particular, neonatal and weaned animals are susceptible to physiological stresses (such as feeding practices, farm management, and dietary needs), which can lead to the invasion of pathogenic bacteria, potentially interfering with the composition of commensal microbes in the gastrointestinal (GI) tract (Gaggìa et al. 2010; Yang et al. 2015). Gut microbial dysbiosis is associated with various diseases and growth retardation in young animals (Chaucheyras-Durand and Durand 2010). In the 1940s, therefore, antibiotics were widely used, not only for the prevention of bacterial infections‚ but also for the improvement of production efficiency since the growth-promoting effects of sub-therapeutic levels of antibiotics were revealed (Stokstad et al. 1950). However, the indiscriminate use of the antibiotics led to the occurrence of antibiotic-resistant pathogenic bacteria and the diffusion of resistance genes from animals to humans (Berends et al. 2001). Consequently, the use of antibiotics as growth promoters (AGPs) in animal production has been prohibited in the European Union since 2006 (EC 2001). Instead, there has been increased focus on the development of alternatives to AGPs as feed additives, such as probiotics, prebiotics, fermented liquid feed, essential oils, and organic acids.

Pigs are an important, and economically significant, source of livestock in the world food market, with 2 billion pigs being supplied each year. The pig industry has been representative of battery farming and AGP use; however, in recent times, alternative nutritional strategies and feed additives have been explored (Pluske 2013). As a result, after the prohibition in 2006, the ratio of average loss to total distribution worldwide has been decreased without the use of AGPs (USDA-FAS, PSD Online). Probiotics, one of the promising alternatives to AGPs in livestock, including pigs, have been reported to have growth-promoting effects that enhance animal production by increasing feed intake, the feed conversion rate, and total body weight (Taras et al. 2007; Chaucheyras-Durand and Durand 2010). In addition, probiotics aid digestive processes in animals by supporting the absorption of certain essential nutrients (Yu et al. 2008).

In the present study, we had a dual objective, to compare in vitro models of human- and pig-targeted GI tract environments and intestinal epithelium, then to select and develop a putative probiotic strain for use as a porcine feed additive. The requirements for effective probiotics to be used in livestock are that they are of host-derived origin, show antimicrobial activity against pathogenic bacteria, exhibit resistance and survival within GI tracts, and are able to adhere to intestinal epithelial cells (de Lange et al. 2010). However, many of the probiotic strains previously used as AGP alternatives were isolated from humans and verified by human-based in vitro screening methods, which were insufficient to reflect the physiology, immune system, and GI microbial community of the host animal (Vlasova et al. 2016; Gaggìa et al. 2010; de Lange et al. 2010). There is therefore a need for a host target-specific probiotic strain, screened by appropriate in vitro methods, that would potentially show enhanced in vivo efficacy when administered to livestock as a feed additive. The genus *Lactobacillus* is a strong candidate as probiotic bacteria. This organism is a significant commensal of the normal gut microbiota of mammals and predominant at the early stage of pig gut microflora construction (Naito et al. 1995; Richards et al. 2005). One of the dominant species, *Lactobacillus salivarius*, frequently isolated from the GI tracts of humans and pigs‚ has been reported as a putative probiotic species due to its ability to exhibit antimicrobial activity, produce short chain fatty acids, attenuate inflammatory conditions, and modulate gut microbiota (Neville and O’Toole 2010; Messaoudi et al. 2013). Therefore, in this study, we investigated the probiotic properties and functional characteristics of *Lactobacillus salivarius* strains isolated from fed pig feces for their use as porcine feed additives.

## Materials and methods

### Strains and culture conditions

Bacterial strains were initially isolated from fed pig feces by analysis of the physiological traits of lactic acid bacteria (i.e., Gram-positive, catalase negative, and rod shaped). The isolated strains were then further identified using the API 50 CHL kit (BioMérieux, Marcy l’Etoile, France) and 16S rRNA sequencing. The amplification of 16S rRNA gene of the strains was conducted with two reactions by PCR using primers 27F 5′ (AGA GTT TGA TCM TGG CTC AG) 3′ and 1492R 5′ (TAC GGY TAC CTT GTT ACG ACT T) 3′. The PCR products were sequenced bi-directionally at Macrogen Corporation (Seoul, Korea). Five representative colonies, all identified as *Lactobacillus salivarius*, were selected based on the fact that they showed comparatively high growth rates. Additionally, *Lactobacillus rhamnosus* GG (ATCC 53103), which originated from a healthy human intestine, was used as a positive control. The pure cultures were maintained in de Man, Rogosa and Sharpe (MRS) broth (Difco, Detroit, MI, USA) and stored at −80 °C in MRS broth with 20 % (*v*/*v*) sterile glycerol. Experiments performed under anaerobic conditions used an anaerobic chamber (Coy Laboratory Products, Ann Arbor, MI, USA) with an atmosphere consisting of 5 % CO_2_, 10 % H_2_, and 85 % N_2_.

### Susceptibility to antibiotics

The minimum inhibitory concentrations (MICs) for selected antibiotics were determined using a modification of the broth microdilution susceptibility method for *Lactobacillus* spp. specified in the National Committee for Clinical and Laboratory Standards Institute guidelines (NCCLS 2010). All strains were incubated for 18 h at 37 °C, and then diluted to an optical density of 0.3 at 600 nm with a double-enriched concentration of MRS medium. Double concentrations of the antimicrobial agents were added to a 96-well microplate as a two-fold serial dilution. The microbiological cut-off values (mg/L) of antibiotics were derived from the European Food Safety Authority guidelines (EFSA 2012).

### Biogenic amine production, hemolytic and gelatinase activity

According to the method of Bover-Cid and Holzapfel (1999), biogenic amine production was analyzed in the isolated strains for 4 days at 37 °C under aerobic and anaerobic conditions. *Enterococcus faecalis* ATCC 29212 was used as a positive control. The hemolytic and gelatinase activities of the strains were also determined (Birri et al. 2013). *Staphylococcus aureus* ATCC 25923 and *Enterococcus faecalis* ATCC 29212 were used as positive controls, respectively.

### Fermentative and enzymatic profiling

Carbohydrate fermentation and metabolic end products were determined using API 50 CHL and API ZYM kits (BioMérieux), respectively. Strains were prepared at 2.0 McFarland concentrations, inoculated into API 50 CH test strips, and incubated at 37 °C for 48 h. For enzymatic profiling, all strains were diluted to 5.0–6.0 McFarland with API suspension medium, inoculated into API ZYM test strips, and then incubated at 37 °C for 4 h. After the addition of one drop of ZYM reagent, A and B were serially added to each strip and incubated for 5 min, allowing colorimetric analysis to be performed.

### Acid and bile tolerance

All strains were propagated in MRS broth for 16 h at 37 °C under anaerobic conditions. The cultures were washed and inoculated into 30 ml of MRS broth with the pH adjusted to 2, 2.5, 3, 3.5, and 4 with 1 N HCl. The samples were incubated at 37 °C under anaerobic conditions for 2 h, and the number of viable (cfu ml^−1^) were counted and compared with the initial counts (Hyronimus et al. 2000). Bile tolerance of the *Lactobacillus* strains was determined by inoculating 5 % of the washed cells into 30 ml of MRS broth supplemented with 0.1, 0.2, 0.3, and 0.5 % (*w*/*v*) bovine bile (Sigma-Aldrich, St. Louis, MO, USA) and incubating at 37 °C anaerobically for 3 h. The optical densities were detected every hour (Raghavendra and Halami 2009).

### BSH activity

Bile salt deconjugation activity was screened using a previously reported method (du Toit et al. 1998) with some modifications. Test strains were incubated for 18 h and spotted onto MRS agar supplemented with 0.5 % (*w*/*v*) sodium salt of taurodeoxycholic acid (Sigma) and 0.37 g l^−1^ CaCl_2_. Plates were incubated at 37 °C for 3 days in an anaerobic chamber. The bile salt hydrolase (BSH) activity was semiquantified by measuring the diameter of the precipitation zones. This assay was conducted in duplicate.

### Antimicrobial activity

The antimicrobial activity of the strains was determined using the modified agar diffusion method described by Schillinger and Lücke (1989). The indicator stains used in this test were *Escherichia coli* K88, *Enterococcus faecalis* ATCC 29212, *Listeria monocytogenes* KCTC 13064, and *Listeria innocua* ATCC 33090. Briefly, stationary phase cells of the *Lactobacillus* and indicator strains were collected by centrifugation at 9500×*g* for 10 min. The cells were washed and diluted to a density of approximately 1 × 10^8^ cfu ml^−1^. The pathogens were spread on MRS agar plates using a sterilized swab. Sterilized disks were placed in the plates using a disk dispenser (Oxoid, Cambridge, UK), and 10 μl of *Lactobacillus* cell suspension was inoculated onto the disk. After incubation for 24 h at 37 °C under aerobic or anaerobic conditions, the diameter of the zone of inhibition was measured in centimeters.

### Inhibition of pathogen adherence to PSI cells

The pig small intestinal cell line PSI was kindly provided by Professor Dr. Avrelija Cencič (Department of Microbiology, University of Maribor, Slovenia). PSI cells were seeded at a density of 1 × 10^5^ cells/cm^2^ per 12-mm membrane Transwell insert with a seeding area of 1.12 cm^2^ and a pore size of 0.4 μm (Corning, Acton, MA, USA). The enterotoxigenic *Escherichia coli* (ETEC) K88 was obtained from the culture collection of the College of Veterinary Medicine Laboratory, Seoul National University, South Korea. *Escherichia coli* K88 was co-cultured with *Lactobacillus* strains to observe both the change in the transepithelial electrical resistance (TEER) and bacterial adherence on PSI cells. The *Lactobacillus* strains and *Escherichia coli* K88 were propagated at a concentration of 1 × 10^7^ and 5 × 10^6^ cfu ml^−1^, respectively. Then, 250 μl of each *Lactobacillus* strain was mixed with the same volume of ETEC K88 (total volume 0.5 ml), and the mixtures were inoculated onto pregrown PSI Transwell inserts. During incubation at 37 °C in a 5 % CO_2_ atmosphere, the TEER was detected using a Millicell-ERS electrode (Millipore, Bedford, MA, USA) hourly, and three wells for each sample were washed and detached from the Transwell after 2.5 h. The adherence assay was conducted as described for the adhesion method below (Anderson et al. 2010). To differentiate and enumerate *Lactobacillus* strains and *Escherichia coli* K88, MRS and Salmonella-Shigella agar (Difco) were used selectively.

### In vitro human and pig GIT simulation models

The gastrointestinal tract (GIT) model consists of three digestive compartments divided into the mouth, stomach, and small intestine. The pig GIT simulation model was based on the human model (Oomen et al. 2003) with modifications to the mucin concentration, pH, and incubation time, to optimize it more closely to the physiological traits of pigs (Kararli 1995; Merchant et al. 2011). The modified factors and the constituents and concentrations of the various synthetic juices are listed in Table 1. In addition, each artificial juice was mixed and preheated to 37 °C just before use, and all chemicals were purchased from Sigma-Aldrich. To simulate the in vitro digestion of humans and pigs, 7 ml of the *Lactobacillus salivarius* strains (1 × 10^9^ cfu ml^−1^) were centrifuged and resuspended in 1 ml of 1× phosphate buffered saline, then mixed with 6 ml of saliva (pH 6.5). After 5-min incubation, 12 ml of gastric juice was added and incubated for 2 h. Then, 12 ml of duodenum juice, 6 ml of bile acid, and 2 ml of NaHCO_3_ were added, followed by further incubation for 2 and 5 h, to reflect the time taken for ingesta to pass through human and pig intestines, respectively. Incubation was performed at 37 °C in an anaerobic chamber and centrifuged at 50 rpm to simulate peristaltic contractions. Test strains were harvested at three time points to simulate the mouth, stomach, and small intestine. The harvested cells were serially diluted and plated onto MRS agar. Experiments were carried out in triplicate and repeated three times.

**Table 1:**
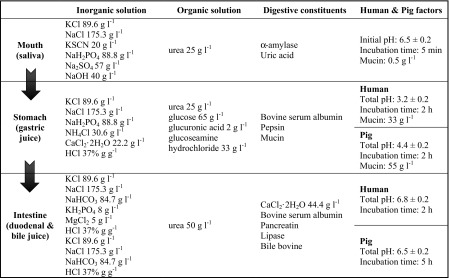
The constituents, chemicals, and conditions used for the human and porcine in vitro gastrointestinal tract (GIT) models

### Adhesion ability to Caco-2 and PSI cells

Caco-2 and PSI cells were grown on Dulbecco’s modified Eagle’s medium (DMEM, Gibco, Carlsbad, CA, USA) supplemented with 10 % fetal bovine serum (FBS, Gibco), l-glutamine (2 mmol l^−1^), 100 μg streptomycin ml^−1^, 100 U penicillin ml^−1^, and 0.25 μg amphotericin B ml^−1^ (Gibco) at 37 °C in a 5 % CO_2_ atmosphere. For the adhesion assay, cells were seeded in 12-well plates at a concentration of 2.5 × 10^6^ cfu ml^−1^ and incubated until confluence for 21 days prior to the assay. The cell culture medium was changed on alternate days with preheated fresh medium, and the cells were washed with DMEM without antibiotics prior to use.

Overnight cultures of the *Lactobacillus salivarius* strains grown in 90 % DMEM supplemented with 2 % FBS and 10 % MRS were harvested, resuspended with preheated DMEM without antibiotics, and adjusted to an optical density of 1.0 at 600 nm (approximately 1 × 10^8^ cfu ml^−1^). A 1-ml aliquot of the bacterial DMEM culture was inoculated into each well of the cell culture plate. The plates were incubated at 37 °C for 90 min in a 5 % CO_2_ atmosphere. Then, each well was washed twice with 1 × phosphate buffered saline and cell layers with attached bacteria were lysed with 1 ml of 0.1 % Trypsin-EDTA solution (Gibco). The number of viable bacterial cells was enumerated using the serial dilution method from the initial and the detached lysates on MRS agar (Schillinger et al. 2005). Experiments were carried out in triplicate and repeated three times.

### Statistical analysis

Data were analyzed using the Student’s *t* test to evaluate differences in discrete variables between the samples.

### Accession numbers

The nucleotide sequences of 16S rRNA genes have been deposited at the GenBank database under the accession numbers KX266895-KX266899 for the representative five strains studied; *Lactobacillus salivarius* strains LS1, LS3, LS4, LS6, and LS8. *Lactobacillus salivarius* strain LS6 isolated from fed pig feces has been deposited at Korean Collection for Type Cultures (KCTC), Jeollabuk-do, South Korea, under the accession number KCTC 18458P.

## Results

### Characterization of probiotic properties

#### Safety assessment

The MIC values of the five selected strains were screened for seven antibiotics. Based on the overall biological breakpoints, strains LS6 and LS8 were found to be susceptible to all of the antibiotics tested, except vancomycin. On the other hand, resistance to erythromycin was detected in strains LS3 and LS4, and resistance to clindamycin was detected in strains LS1, LS3, and LS4 (Table 2). None of the isolates exhibited biogenic amine production or hemolytic activity. In addition, gelatinase activity in the test strains was lower than in the positive control, with the exception of strain LS1 under anaerobic conditions (Table 3).

**Table 2:** Minimum inhibitory concentrations (μg ml^−1^) of antibiotics to *Lactobacillus salivarius* strains

	AMP	VAN	GEN	CHL	STR	ERY	CLI
*L. salivarius*							
LS1	2	>512	64	2	128	1	2
LS3	2	>512	64	4	256	>8	>8
LS4	1	>512	128	4	512	>8	>8
LS6	1	>512	128	2	256	1	0.5
LS8	2	>512	128	2	256	1	0.25
Suggested breakpoints for *L. salivarius*							
NCCLS		≥16	≥16			≥8	≥2
EFSA	4	n.r.	16	4	64	1	1
SCAN	2	4	1	16	16	4	
Danielsen and Wind (2003)	4	4	256	16	>256	1	2

*AMP* ampicillin, *VAN* vancomycin, *GEN* gentamicin, *CHL* chloramphenicol, *STR* streptomycin, *ERY* erythromycin, *CLI* clindamycin, *n.r.* not required

**Table 3:** Safety assessment of the metabolic end products of the *Lactobacillus salivarius* test strains to be used as probiotics

Bacterial culture	Biogenic amine production		Hemolytic activity		Gelatinase activity^a^	
	AR	ANAR	AR	ANAR	AR	ANAR
Positive control						
*E. faecalis* ATCC 29212	+	+	n.r.	n.r.	1.1 ± 0.0	1.0 ± 0.0
*S. aureus* ATCC 25923	n.r.	n.r.	+	+	n.r.	n.r.
*L. salivarius*						
LS1	−	−	−	−	0.9 ± 0.1	1.0 ± 0.0
LS3	−	−	−	−	0.9 ± 0.0	0.8 ± 0.0
LS4	−	−	−	−	0.8 ± 0.0	0.8 ± 0.0
LS6	−	−	−	−	0.8 ± 0.0	0.8 ± 0.0
LS8	−	−	−	−	0.7 ± 0.0	0.7 ± 0.0

*n.r.* not required, *AR, ANAR* 37 °C aerobic and anaerobic conditions, respectively

^a^Average diameter (cm) values ± the standard deviation (*n* = 2)

#### Metabolic profiles

**Fig. 1:**
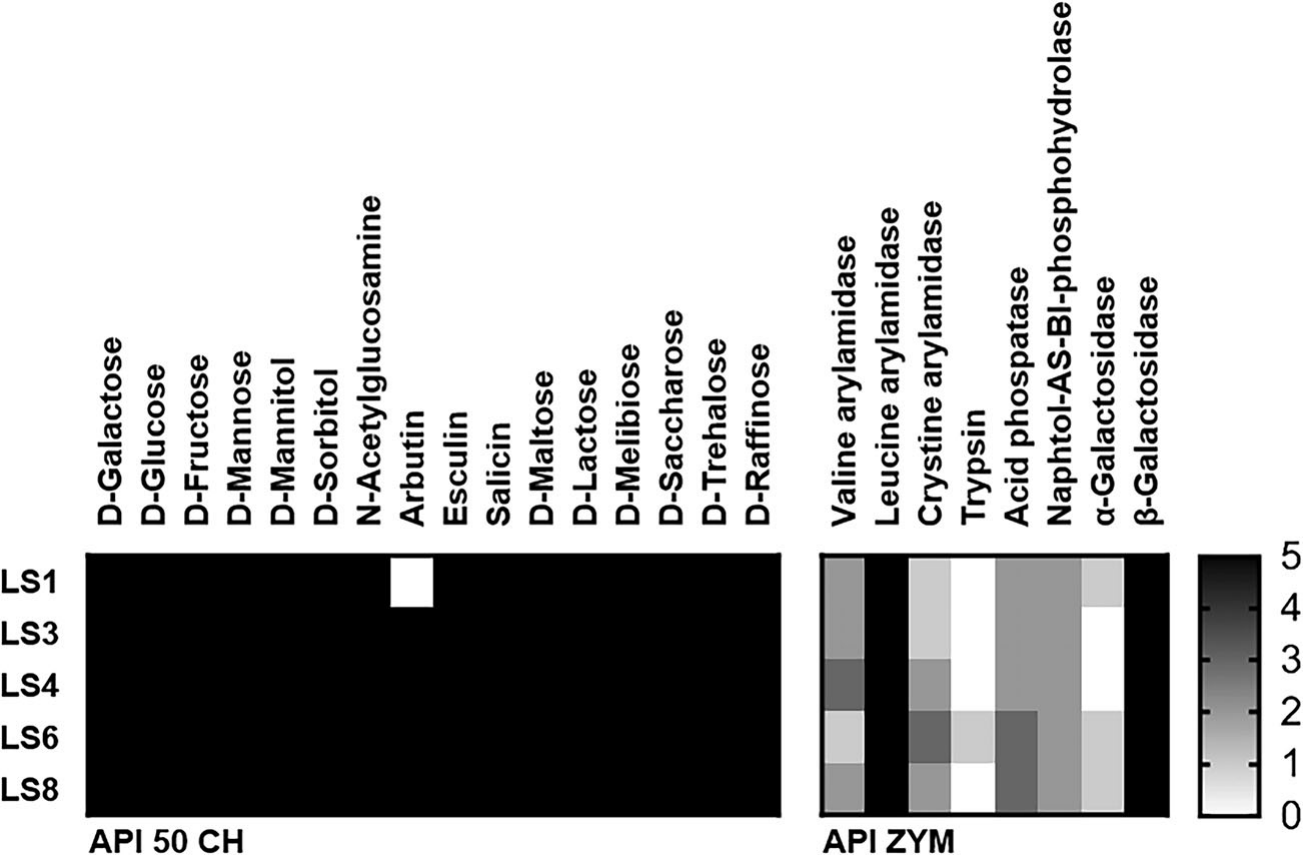
Fermentative and enzymatic profiling of selected *Lactobacillus salivarius* strains isolated from pig feces. The colorimetric intensity is indicated by *color gradients*: *black* represents high activity, while *white* represents no reaction. The carbohydrates and enzymes for which most strains did not react are not shown

The pig-derived *Lactobacillus salivarius* strains showed identical patterns of fermentation, with the exception of strain LS1 that did not use arbutin (Fig. 1). On testing enzymatic activity, all strains showed strong leucine arylamidase and β-galactosidase activity and moderate activities for six other enzymes (Fig. 1).

#### Acid and bile tolerance, bile salt hydrolase activity, and antimicrobial activity

All five *Lactobacillus salivarius* strains demonstrated tolerance to acidic conditions (i.e., pH 3). After 2 h of incubation in MRS medium at pH 3, the survival rates of *Lactobacillus salivarius* strains LS3 and LS6 were 85.88 and 95.17 %, respectively. The LGG positive control showed a survival rate of 77.3 %. Although all of the test isolates showed lower growth rates than LGG (only 30.4 % of the slope) in MRS medium supplemented with 0.3 % (*w*/*v*) bovine bile, they were able to grow under conditions of bile salt stress (Table 4). In addition, the *Lactobacillus salivarius* strains exhibited high BSH ability, as shown by precipitation zones in the BSH agar plate (Table 4).

**Table 4:** Probiotic properties of *Lactobacillus* strains

Bacterial culture	Acid tolerance^a^ (%)		Bile tolerance (% slope^b^)	BSH activity	Diameter of the zone of inhibition^c^							
					E. c		E. f		L. m		L. i	
	0 h	2 h			AR	ANAR	AR	ANAR	AR	ANAR	AR	ANAR
*L. salivarius*												
LS1	100	66.95^*^	18.4	+	+	++	(+)	−	++	−	++	+++
LS3	100	85.88	6.6	+	+	+	−	−	++	−	+	++
LS4	100	50.11^**^	12.0	+	+	++	−	−	++	−	++	++
LS6	100	95.17^*^	15.8	+	+	++	−	−	++	−	+++	++
LS8	100	59.29^*^	10.8	+	+	++	(+)	−	++	−	++	++
*L. rhamnosus*												
GG	100	77.30	30.4	−	+	++	−	−	++	−	++	++

*L. rhamnosus* GG, a commercial lactic acid bacteria, was used as a standard reference culture

*E. c Escherichia coli* K88, *E. f Enterococcus faecalis* ATCC 29212, *L. m Listeria monocytogenes* KCTC 13064, *L. i Listeria innocua* ATCC 33090, *AR, ANAR* 37 °C aerobic and anaerobic conditions, respectively, *BSH* bile salt hydrolase

^a^Cell survival at pH 3.0. Significance is indicated as follows: ^*^ *P* ≤ 0.05; ^**^ *P* ≤ 0.01, compared to the survival of LGG

^b^Slope; (x, y) = (time, OD_600_ value) of 0–3 h in MRS broth supplemented with 0.3 % bovine bile. *r* ^*2*^ = 0.97 ± 0.02

^c^+++, >3 cm; ++, >2 cm; +, >1 cm; (+), < 1 cm; −, no clear zone

The results of the agar diffusion assay demonstrated the antagonistic activities of the washed cells of all of the test strains against *Escherichia coli* K88 and *Listeria innocua* ATCC 33090 under both aerobic and anaerobic conditions, whereas the strains only inhibited the growth of *Listeria monocytogenes* KCTC 13064 under aerobic conditions. None of the test strains exhibited a significant inhibitory effect on *Enterococcus faecalis* ATCC 29212 (Table 4). Bacteriocin production was also investigated using cell-free supernatant adjusted to pH 6.5 with 1 N NaOH, but no bacteriocin production was detected in any of the *Lactobacillus salivarius* strains. Moreover, cell-free supernatant that was not pH-adjusted showed no significant antibacterial effect on any other indicator strains (data not shown).

### Functionality in host target-specific models

#### Protection of epithelial cells

In a previous experiment, all *Lactobacillus salivarius* isolates were screened in a TEER assay, and isolates LS4, LS6, and LS8 were selected as having a positive effect on TEER measurements (Fig. S1, Supporting Information). *Lactobacillus rhamnosus* GG also showed a significant increase in the TEER value to 172 % after 6-h incubation.

**Fig. 2:**
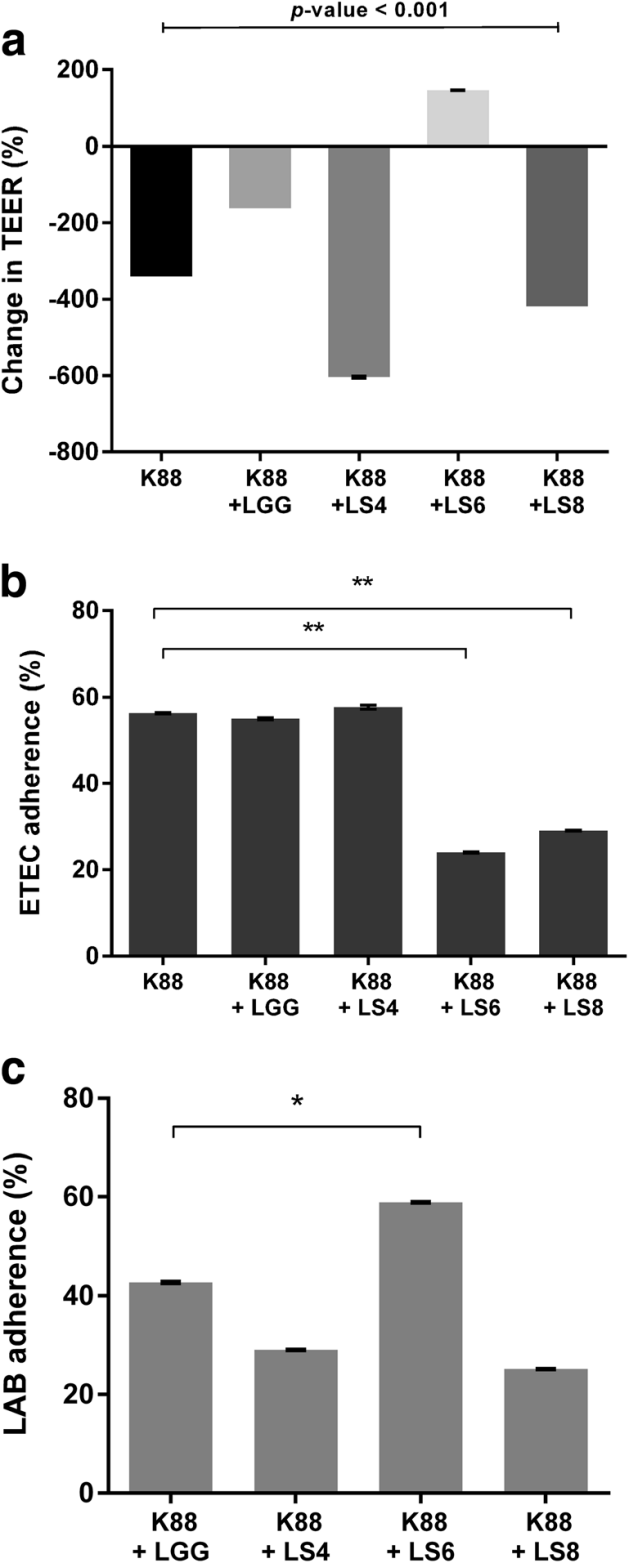
Change in TEER across confluent PSI cells and bacterial adherence to PSI cells during co-culturing of *Lactobacillus* strains and enterotoxigenic *Escherichia coli* (ETEC) K88. The *graphs* (**a**) represent TEER values (% of initial value, 0 h) of PSI cells after 6 h of co-incubation of *Lactobacillus* strains and *Escherichia coli* K88. Adherence was investigated 2.5 h after bacterial treatment. **b** Binding ability of ETEC K88 to PSI cells. **c** Binding ability of human-derived LGG strain and pig-derived *Lactobacillus salivarius* strains. The data represent the means and standard errors of three replicates. Significance is indicated as follows: *P ≤ 0.05; **P ≤ 0.01

The co-culture of *Escherichia coli* K88 and test strain LS6 significantly increased the TEER value to 146 %, whereas co-incubation of *Escherichia coli* K88 with other *Lactobacillus* strains, even with LGG, reduced the TEER value of PSI cells (Fig. 2a). After 2.5 h of treatment with ETEC K88 and test strain LS6, ETEC showed an adherence rate of 24 % compared with 58.9 % for strain LS6 strain (Fig. 2b, c). ETEC K88 binding was not affected by either the LGG or LS4 test strains (Fig. 2b), and the TEER of PSI cells was significantly reduced by co-culturing ETEC with LS4 (Fig. 2a). Strain LS8 reduced ETEC adherence to 29 % (Fig. 2b), with LS8 adherence being even lower at 25.1 % (Fig. 2c); moreover, disruption of the integrity between PSI cells was observed by a change in the TEER value (Fig. 2a).

#### Survival in the GIT

**Fig. 3:**
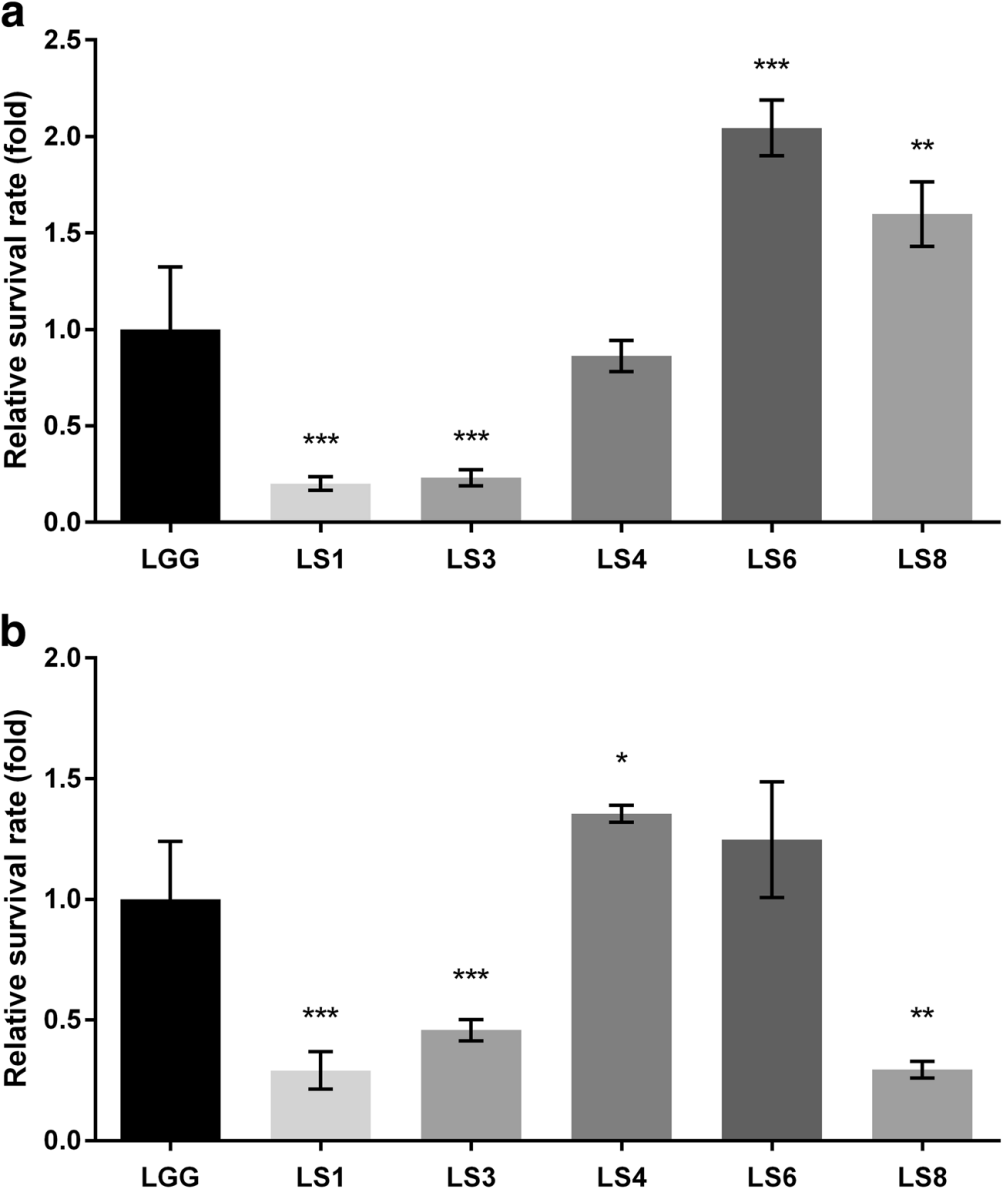
The viability of *Lactobacillus* strains on passing through the in vitro GIT model, simulating **a** the human and **b** the pig GI tract. Relative survival rate was calculated as a fold change compared with LGG survival. See also Table 1 for information on the factors modified between the porcine and human GIT models. The data represent the means and standard errors of three replicates. Significance is indicated as follows: *P ≤ 0.05; **P ≤ 0.01; ***P ≤ 0.001

The simulated pig GIT model was based on the human GIT model with modifications in the pH, incubation time, and amount of mucin (Table 1). All strains showed strain-specific survival compared with the control (strain LGG) in both GIT models (Fig. 3). Strains LS1 and LS3 showed lower survival rates than did control strain LGG under both GIT models, whereas strain LS4 showed a survival rate increase of about 0.86-fold in the human GIT model compared with the 1.4-fold in the pig GIT model. By contrast, the survival of strain LS8 was increased 1.6-fold and 0.3-fold in the human and pig GIT models, respectively. *Lactobacillus salivarius* LS6 exhibited high tolerance and adaptation in response to both GITs but was more sensitive to pig GIT stress (Fig. 3b).

#### Adhesion ability to epithelial cells

**Fig. 4:**
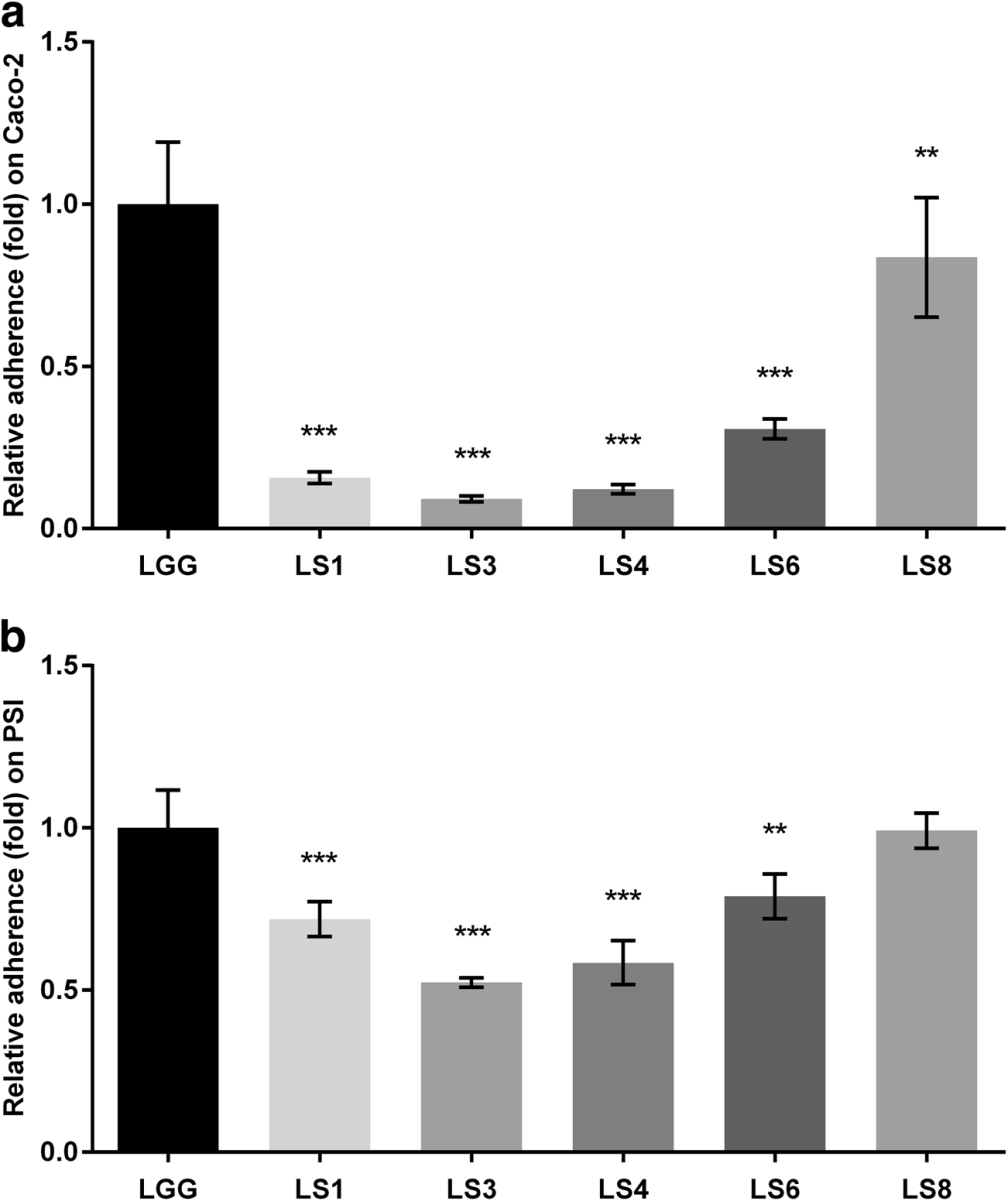
The relative adhesion ability of *Lactobacillus* strains on **a** Caco-2 and **b** PSI cells. Relative adherence was calculated as a fold change compared with LGG adherence, which was 5.1 and 15.5 % on Caco-2 and PSI cells, respectively. The data represent the means and standard errors of three replicates. Significance is indicated as follows: **P ≤ 0.01; ***P ≤ 0.001

The adhesion ability of all strains was similar to each cell line compared with control strain LGG, which showed 5.1 and 15.5 % adhesion to Caco-2 and PSI cells, respectively (Fig. 4a, b). With the exception of strain LS8, all of the test strains were observed to be lower than 0.3-fold (1.6 % adherence) to Caco-2 cells and at least 0.5-fold (8 % adherence) to PSI cells, compared with the control strain LGG. The relative adhesion ability of strain LS8 was similar to that of control strain LGG to both Caco-2 and PSI cells.

## Discussion

In the present study, we identified the probiotic properties of *Lactobacillus salivarius* strains isolated from fed pig feces, which could potentially be used as feed additives for porcine. Recently, increased research into the development of probiotics for humans and animals confirmed that probiotic action may be influenced by the host (Vlasova et al. 2016). Therefore, we developed and evaluated pig-based screening models that differ from existing human-based methods. Differences were observed in the viability and adhesive ability of strains under porcine and human conditions.

Prior to these studies, isolates have been assessed for their safety and functionality as probiotics. One of the safety concerns regarding probiotics is the existence of antibiotic resistance. According to the National Committee for Clinical and Laboratory Standards Institute guidelines (NCCLS 2010) and the European Food Safety Authority (EFSA 2012) breakpoints, five *Lactobacillus salivarius* strains isolated from pig feces were found to be resistant to the antibiotics tested, with the exception of ampicillin (Table 2). In addition, when compared with the microbiological breakpoints proposed by the Scientific Committee for Animal Nutrition (SCAN 2002), all isolates showed high MIC values for vancomycin, gentamicin, and streptomycin. However, some *Lactobacillus* spp. are considered to be intrinsically resistant to vancomycin (NCCLS 2010), and there are no well-defined standards for the MIC ranges and the MIC determination methods for *Lactobacillus* spp. (SCAN 2002). Moreover, the MIC values of microorganisms depend on the type of test medium used. In this study, MRS medium was employed because the five isolates tested could not grow in cation-adjusted Mueller-Hinton II broth supplemented with 2.5 % (*v*/*v*) lysed horse blood, the standard medium used for *Lactobacillus* spp. as suggested by the NCCLS (2010), EFSA (2012), and SCAN (2002). Therefore, breakpoints suggested by the NCCLS, EFSA, and SCAN were insufficient to determine the susceptibility of these strains, so MIC breakpoints based on MRS medium, as suggested by Danielsen and Wind (2003), were additionally applied. As a result, it could be considered that *Lactobacillus salivarius* strains LS6 and LS8 are safe against antibiotic resistance transfer for use in livestock.

Biogenic amines are nitrogenous substances, mainly produced by the decarboxylase of lactic acid bacteria during improper fermentation. The risk of biogenic amines to humans has been reported in several studies (Bover-Cid and Holzapfel 1999; Ladero et al. 2010; EFSA 2011). However, there is little information regarding the influence of biogenic amines on pig health, but it has been reported that high levels of biogenic amines in liquid feed reduced feed intake and growth performance of pigs (Pedersen 2001; Brooks 2003). According to our result, the pig-derived strains did not produce biogenic amines from the four amino acids tested (Table 3), and thereby appear safe for use as additives to porcine feed containing high levels of protein. Furthermore, none of the tested isolates indicated hemolytic or gelatinase activity perceived as potential virulence factors in food and feed (Table 3) (Oakey et al. 1995; Bernardeau et al. 2006).

Potentially detrimental enzyme activities are another important safety issue to be considered regarding probiotics, in particular β-glucosidase and β-glucuronidase are known to be carcinogenic enzymes in the intestine, whereas β-galactosidase is beneficial for the relief of lactose intolerance in animals (Lee et al. 2008; Bujnakova et al. 2013). The *Lactobacillus salivarius* strains tested in this study showed high β-galactosidase activity (Fig. 1).

According to Lähteinen et al. (2010), *Lactobacillus salivarius* isolates originating from porcine intestines were generally intolerant to pH 2. Our *Lactobacillus salivarius* strains could survive at pH 3 (Table 4), and *Lactobacillus salivarius* LS6 was even able to survive at a rate over 55 % at pH 2 (data not shown).

Bile acids are metabolic end products synthesized from cholesterol in the liver that are conjugated with glycine or taurine and then secreted into the duodenum through the gall bladder at an average daily volume of 400–800 mg (Tsai et al. 2011). The conjugated bile acids can dissolve bacterial membranes, thus to survive bacteria need to tolerate high levels of bile acids in the GI tract. We found that *Lactobacillus salivarius* strains were able to tolerate and grow at 0.5 % bile salt concentrations (data not shown). Furthermore, deconjugation activity was shown for all *Lactobacillus salivarius* strains in a strain-specific manner, whereas no BSH activity was observed for *Lactobacillus rhamnosus* strain GG (Table 4). Conjugated bile acids in the small intestine can be deconjugated and converted to secondary bile acids by bacterial BSH enzyme, which is related to the host serum cholesterol-lowering effect of the enterohepatic circulation (Corzo and Gilliland 1999). Thereby, the BSH activity of probiotics can contribute to the health benefits of animal products.

Enterotoxigenic *Escherichia coli* (ETEC) K88 is a major enteropathogen in neonatal and weaned pigs, and their fimbrial adherence and production of enterotoxins within the pig intestine can cause diarrheal illness and even death (Francis 2002). All of the strains tested in this study inhibited the growth of ETEC K88 under both aerobic and anaerobic conditions, whereas they inhibited the growth of *Listeria monocytogenes* KCTC 13064 under aerobic conditions only (Table 4). This antagonistic effect was confirmed in the pig small intestine cell line PSI (Fig. 2). TEER and adhesion assays indicated that when the tested lactobacilli were co-cultured with ETEC K88, they inhibit ETEC K88 adherence as well as attenuating disruption of the tight junctions between PSI cells. Tight junctions are one of the mechanisms for sustaining gut epithelium cell-cell adherence. Enteric infections by bacteria such as *Salmonella* spp., *Shigella* spp., enterohemorrhagic *Escherichia coli*, and ETEC, lead to the breakdown of tight junctions by type III secretion systems and bacterial surface proteins, leading to increased cell permeability and the penetration of bacteria across host tissues (Ashida et al. 2011). Many reports suggest that probiotic bacteria could not only improve the intestinal barrier function (Anderson et al. 2010) but also ameliorate the invasion of enteropathogenic *Escherichia coli* and ETEC (Lodemann et al. 2015). In particular, weaned piglets commonly show symptoms of gastroenteritis caused by ETEC K88, and pathogenicity was alleviated by the use of probiotics as feed additives (Setia et al. 2009; Bhandari et al. 2010). Based on our findings, the antipathogenic effect was related to competition for adherence between *Lactobacillu*s strains and ETEC. After co-culturing, the adherence of ETEC K88 was inhibited by *Lactobacillu*s *salivarius* strains LS6 and LS8 (Fig. 2b), but strain LS8 did not adhere well compared with strain LS6 (Fig. 2c). The results of TEER assays indicated that this difference was caused by strain-specific effects on the integrity of tight junctions (Fig. 2a). Therefore, *Lactobacillu*s *salivarius* LS6 may be effective in the treatment of ETEC infection, and the results of this TEER competition assay are useful in the selection of functional probiotics.

Humans and pigs are both monogastric organisms possessing an oral cavity, stomach, and intestine. However, the physiological traits of their GI tracts differ. Merchant et al. (2011) reported that pigs have a longer gut (∼24 m; 2.4 cm/kg body weight) compared with humans (8.9 m; 14 cm/kg body weight). This difference in gut length affects the time taken by ingesta, such as food/feed including probiotics, to pass through the GIT; hence, in our model, we modified the incubation time to simulate the pig intestine. Moreover, there were differences in the average pH values of the stomach and intestine depending on the host (Table 1). In our investigations, when we applied human-based criteria, a commercial probiotic *Lactobacillu*s *rhamnosus* GG was selected as a candidate feed additive, whereas *Lactobacillu*s *salivarius* LS4 was not selected (Fig. 3a). However, *Lactobacillu*s *salivarius* LS4 exhibited the highest survival rates under conditions of pig GI tract stress (Fig. 3b). *Lactobacillu*s *salivarius* LS8 could also be selected (Fig. 3a), even though it showed the lowest survival rates in the pig GIT model (Fig. 3b). These results indicate that human-based stress tolerance screening methods are not always suitable for the development of porcine probiotics. Although there is still scope for improvement, our suggested in vitro porcine GIT simulation model is effective for selecting probiotic candidates for pig-targeted application.

The Caco-2 human colon adenocarcinoma cell line has been widely used in adhesion assays for the identification of potential probiotic lactic acid bacteria. However, these cells do not derive from the small intestine; rather, they are a tumorigenic cell line (Cencič and Langerholc 2010). Therefore, in this study, the pig-derived non-carcinoma small intestine cell line PSI, established by Cencič and colleagues (Gradisnik et al. 2006), was applied. Only *Lactobacillu*s *salivarius* LS8 was considered a probiotic candidate based on the adhesion properties to Caco-2 cells (Fig. 4a); however, all of the pig-derived isolates were considered probiotic candidates based on their adhesion properties to PSI cells (Fig. 4b). These findings highlight the inadequacies of Caco-2 cells as an in vitro adhesion model with which to evaluate the binding ability of strains for porcine.

In conclusion, we identified *Lactobacillu*s *salivarius* strain LS6 as a potential probiotic strain for use as a porcine feed additive. Our findings confirm that potential probiotics should be selected based on target host-specific criteria to ensure their suitability and functionality in the intended host. Furthermore, the scientifically validated in vitro methods presented here can be used to replace animal testing experiments, thereby reducing costs and eliminating unnecessary suffering in line with the Registration, Evaluation and Authorization of Chemicals (REACH) regulations.

## Electronic supplementary material

ESM 1 (PDF 55 kb)
