# Supplementary material for: Development of putative probiotics as feed additives: validation in a porcine-specific gastrointestinal tract model
Source: Appl Microbiol Biotechnol. 2016 Sep 15;100(23):10043–54. doi: 10.1007/s00253-016-7812-1 (PMC5102953; doi:10.1007/s00253-016-7812-1)
Supplement: Supplementary file 1 — (PDF 55 kb) [file 253_2016_7812_MOESM1_ESM.pdf]

Supplementary material

Applied Microbiology and Biotechnology

Development of putative probiotics as feed additives: validation in a porcine-specific gastrointestinal tract model

Soyoung Yeo[1] · Suro Lee[1] · Hyunjoon Park[3] · Heuynkil Shin[4] · Wilhelm Holzapfel[3] · Chul Sung Huh[1,2,*]

* Corresponding author

Tel.: +82-10-9216-9601

Fax: +82-33-339-5717

E-mail: chulsunghuh@gmail.com

1 Research Institute of Eco-friendly Livestock Science, Institute of Green-Bio Science and Technology, Seoul National University, Pyeongchang, Gangwon-do 232-916, South Korea

2 Graduate School of International Agricultural Technology, Seoul National University, Pyeongchang, Gangwon-do 232-916, South Korea

3 Advanced Green Energy and Environment Institute (AGEE), Handong Global University, Pohang, Gyeongbuk 791-708, South Korea

4 School of Life Sciences, Handong Global University, Pohang, Gyeongbuk 791-708, South Korea

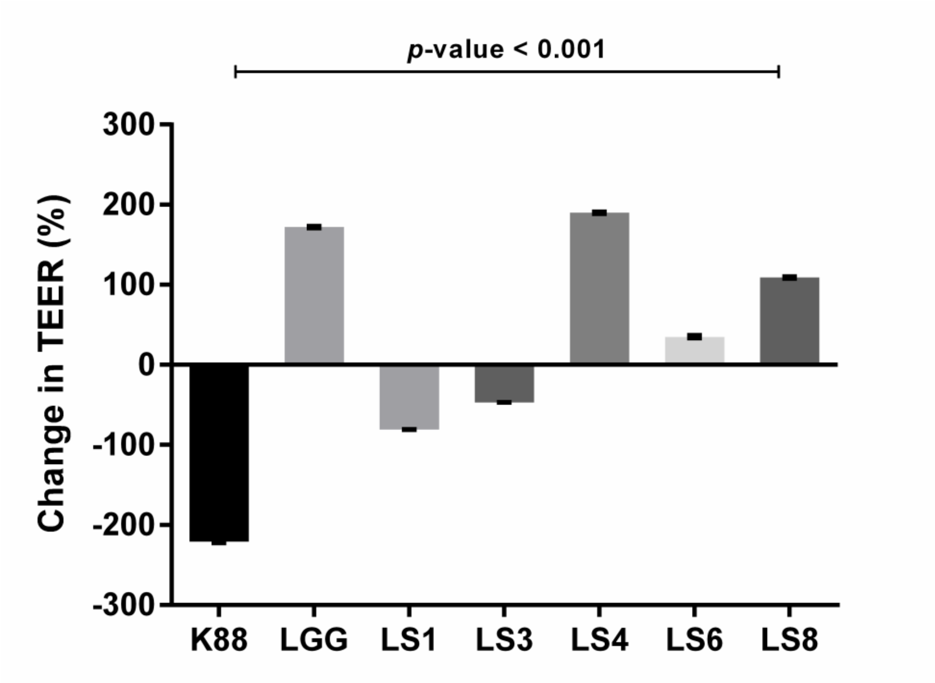

Fig. S1 Change in TEER across confluent PSI cells after treatment with *Lactobacillus* strains or *E. coli* K88 individually. The graph represents the TEER values (% of initial value, 0 h) of PSI cells after 6 h. The data represent the means and standard errors of three replicates
